# Genetic and Epigenetic Causes of Pituitary Adenomas

**DOI:** 10.3389/fendo.2020.596554

**Published:** 2021-01-26

**Authors:** Mengqi Chang, Chengxian Yang, Xinjie Bao, Renzhi Wang

**Affiliations:** Department of Neurosurgery, China Pituitary Disease Registry Center, Peking Union Medical College Hospital, Peking Union Medical College & Chinese Academy of Medical Sciences, Beijing, China

**Keywords:** pituitary adenomas, molecular markers, acromegaly, Cushing’s disease, non-secreting adenomas

## Abstract

Pituitary adenomas (PAs) can be classified as non-secreting adenomas, somatotroph adenomas, corticotroph adenomas, lactotroph adenomas, and thyrotroph adenomas. Substantial advances have been made in our knowledge of the pathobiology of PAs. To obtain a comprehensive understanding of the molecular biological characteristics of different types of PAs, we reviewed the important advances that have been made involving genetic and epigenetic variation, comprising genetic mutations, chromosome number variations, DNA methylation, microRNA regulation, and transcription factor regulation. Classical tumor predisposition syndromes include multiple endocrine neoplasia type 1 (MEN1) and type 4 (MEN4) syndromes, Carney complex, and X-LAG syndromes. PAs have also been described in association with succinate dehydrogenase-related familial PA, neurofibromatosis type 1, and von Hippel–Lindau, DICER1, and Lynch syndromes. Patients with aryl hydrocarbon receptor-interacting protein (*AIP*) mutations often present with pituitary gigantism, either in familial or sporadic adenomas. In contrast, guanine nucleotide-binding protein G(s) subunit alpha (*GNAS*) and G protein-coupled receptor 101 (*GPR101*) mutations can lead to excess growth hormone. Moreover, the deubiquitinase gene *USP8*, *USP48*, and *BRAF* mutations are associated with adrenocorticotropic hormone production. In this review, we describe the genetic and epigenetic landscape of PAs and summarize novel insights into the regulation of pituitary tumorigenesis.

## Introduction

Pituitary adenomas (PAs) are the second most common brain tumors, accounting for approximately 15% of all primary brain tumors (1). PAs can be classified based on the types of hormones that they excessively secrete into the blood. The clinical presentations caused by hormone overproduction in PAs are closely related to the pituitary cell types, as follows: corticotropin-secreting corticotroph adenomas result in Cushing’s disease, growth hormone (GH)-secreting somatotroph adenomas result in acromegaly, prolactin-secreting lactotroph adenomas result in hyperprolactinemia, and thyrotropin-secreting thyrotroph adenomas result in hyperthyroidism (2). Non-secreting adenomas, such as null cell adenomas, silent gonadotroph adenomas, silent corticotroph adenomas, and silent somatotroph adenomas, lead to hypogonadism and often manifest as incidental sellar masses (2). Of the different PA types, only lactotroph adenomas are treated with dopamine agonists as a first-line option. Because of a lack of effective drugs, other PA subtypes are generally treated with transsphenoidal surgery as the first-line therapy (3). Furthermore, because many PAs are invasive and unresectable—or in some cases of Cushing’s disease, because the tumors themselves are too small to be detected or completely removed during surgery—drugs and stereotactic radiosurgery are needed to achieve tumor control or biochemical remission. However, despite current treatments, the 10-year recurrence rate remains as high as 7–12% (3, 4). It is therefore important to obtain a comprehensive understanding of the molecular biological characteristics of different types of PAs, such as gene mutations, DNA methylation, microRNA (miRNA) regulation, and regulation at other levels, to allow the targeted treatment of individuals, thus achieving better prognoses. Herein, we summarize the known variation in the different types of PAs and review the potential molecular targets for future clinical application.

## Syndromic Pituitary Adenoma-Related Variations

Familial PAs can be divided into two types: isolated and syndromic (5). These familial PAs and their molecular mechanisms are described herein.

### Multiple Endocrine Neoplasia Type 1 Syndrome

MEN1 syndrome is classically characterized by the combined occurrence of parathyroid adenomas, PAs (in approximately 30–40% of cases), and neuroendocrine tumors (6). PAs that develop in MEN1 syndrome include lactotroph adenomas (42–62%), silent PAs (15–42%), somatotroph adenomas (6.5–9%), and corticotroph adenomas (3–4%). In addition, somatic mutations in *MEN1* can also be found in sporadic PAs (7). The *MEN1* gene is located on chromosome 11q13.1 and encodes a ubiquitously expressed transcription cofactor of cyclins; *MEN1* also participates in G1/S checkpoint regulation (8, 9). Approximately 10% of all MEN1-related PA cases can be attributed to *de novo* mutations, which are sometimes identified as a mosaicism in the proband (10, 11).

### Multiple Endocrine Neoplasia Type 4 Syndrome

Some patients with MEN1 syndrome harbor no *MEN1* mutation. Instead, cyclin-dependent kinase inhibitor 1B (*CDKN1B*) mutations have been detected in these patients. This syndrome is known as MEN4 syndrome (12). Patients with MEN4 syndrome are prone to developing somatotroph adenomas, and can also develop other types of Pas (13). *CDKN1B* mutations are rarely found in sporadic pituitary tumors (14, 15). *CDKN1B* encodes a cyclin-dependent kinase inhibitor that regulates the cell cycle and mitosis from the G1 to the S phase (13). *CDKN1B*-knockout mice develop various types of tumors, including PAs, and this tumorigenesis is associated with accelerated pituitary cell proliferation (16, 17). *CDKN1B* mutations likely lead to PAs by influencing cell cycle regulation.

### Carney Complex

Carney complex is characterized by endocrine and non-endocrine tumors with spotty skin pigmentation, as well as by cardiac and cutaneous myxomas (18). More than two-thirds of patients present asymptomatic elevations of insulin-like growth factor 1 (IGF-1), GH, and prolactin caused by pituitary hyperplasia, and 10% of patients present with adenomas and symptomatic acromegaly (19). In some cases, Carney complex is caused by an inactivating mutation of the *PRKAR1A* gene, which encodes the type 1-alpha regulatory subunit of protein kinase A (20). In addition, a gain-of-function mutation has been described in the gene encoding the catalytic subunit of protein kinase A, *PRKACB* (21).

### X-LAG Syndrome

X-LAG syndrome is a newly defined syndrome in patients with pituitary gigantism or PA who carry microduplications on chromosome Xq26.3 (22, 23). X-LAG syndrome is generally recognized as an aggressive disease because it is difficult to control the excess GH. Most patients require multiple interventions (both surgical and medical), and subtotal or total hypophysectomy is sometimes necessary. In contrast, radiation therapy is not usually helpful. X-LAG syndrome is probably caused by G protein-coupled receptor 101 (GPR101) overexpression because the *GPR101* gene is located on chromosome Xq26.3. GPR101 is coupled to the stimulatory G protein complex and activates adenylate cyclase, increasing cyclic adenosine monophosphate (cAMP) production. In addition, *GPR101* amplification has also been identified as a germline or mosaic mutation (22).

### Succinate Dehydrogenase-Related Familial Pituitary Adenoma

This “3PAs” syndrome, which combines PA with pheochromocytoma/paraganglioma (PPGL), is sometimes associated with mutations in PPGL-predisposing genes, such as the genes encoding SDH*x* (24, 25). Such mutations occur in *SDHA-D* and *SDHA2F*, among others (24–28). SDH is a multimeric enzyme that binds to the inner membranes of mitochondria. It has a dual role: it serves both as a critical step of the tricarboxylic acid or Krebs cycle, and as a member of the respiratory chain that transfers electrons directly to the ubiquinone pool (25, 27, 29). Pituitary hyperplasia has been reported to develop in a *Sdhb*-knockout mouse model (25).

### Neurofibromatosis Type 1 Syndrome

Rarely, optic pathway gliomas cause high GH levels in NF1, while true PAs are extremely rare. Empty sella syndrome and hypopituitarism may also occur in the context of NF1. Lifelong endocrine follow-up is recommended for all NF1 patients (13). A recent case report described a patient with a heterozygous guanine nucleotide-binding protein G(s) subunit alpha (*GNAS*) R201C mutation in a somatotroph adenoma. This was the first reported rare MEN1-like case of genetically diagnosed NF1 complicated with acromegaly caused by somatotroph adenoma (14).

### Von Hippel–Lindau Syndrome

VHL syndrome is a heritable multisystem cancer syndrome that is caused by germline mutations of the VHL tumor suppressor gene. The incidence of this disorder is as high as 1 in 36,000 live births (15). Patients with VHL syndrome are at risk of developing various benign and malignant tumors of the central nervous system [i.e., pituitary stalk hemangioblastomas (15)], kidneys, adrenal glands, pancreas, and reproductive adnexal organs (16). It has been reported that propranolol can decrease the viability of VHL-related hemangioblastomas and renal cell carcinomas *in vitro*, likely by modulating vascular endothelial growth factor expression and inducing apoptosis (17). However, propranolol treatment for this disease is limited to early clinical trials.

### DICER1 Syndrome

DICER1 syndrome is caused by heterozygous germline mutations in the *DICER1* gene (30). Several cases have been reported of rare infantile-onset pituitary blastoma that were mainly caused by germline mutations in *DICER1 *(31). Recently, corticotroph adenomas have also been identified in this tumor syndrome (31). *DICER1* encodes a cytoplasmic endoribonuclease that processes hairpin precursor miRNAs into short, functional miRNAs that downregulate targeted mRNAs, thereby modulating cellular protein production (32). In addition, specific somatic mutations in the *DICER1* RNase III catalytic domain have been identified in several *DICER1*-associated tumor types (31, 33).

### Lynch Syndrome

Lynch syndrome is a cancer-predisposing syndrome caused by germline mutations in genes involved in DNA mismatch repair (34). Germline mutations in *MLH1* (35) and *MSH2* (34) in the mismatch repair pathway, have been identified in Lynch syndrome patients with aggressive corticotropin-secreting adenomas, although these are isolated case reports. Missense mutations have also been detected in four mismatch repair genes (*MSH5*, *MSH6*, *MLH1*, and *MLH3*) in non-secreting adenomas (3).

## Somatotroph Adenomas

The incidence of somatotroph adenomas is approximately 10 cases per 1 million individuals (2, 36, 37). Somatotroph adenomas are GH-secreting somatotropic tumors that exhibit excessive secretion of GH and IGF-1, causing acromegaly and abnormal growth of bones, tissues, and organs in patients. Currently, treatment methods are limited to surgery, radiotherapy, somatostatin receptor (SSTR) ligands, and GH receptor antagonists. Each of these treatments has specific side effects, and the efficacy varies greatly among different patients; thus, it is hard to directly target and inhibit the continuous secretion of GH in postoperative patients (2). It is necessary to further understand the molecular mechanisms of somatotroph adenomas to elucidate new therapeutic targets.

### Genetic Variations

**Figure 1:**
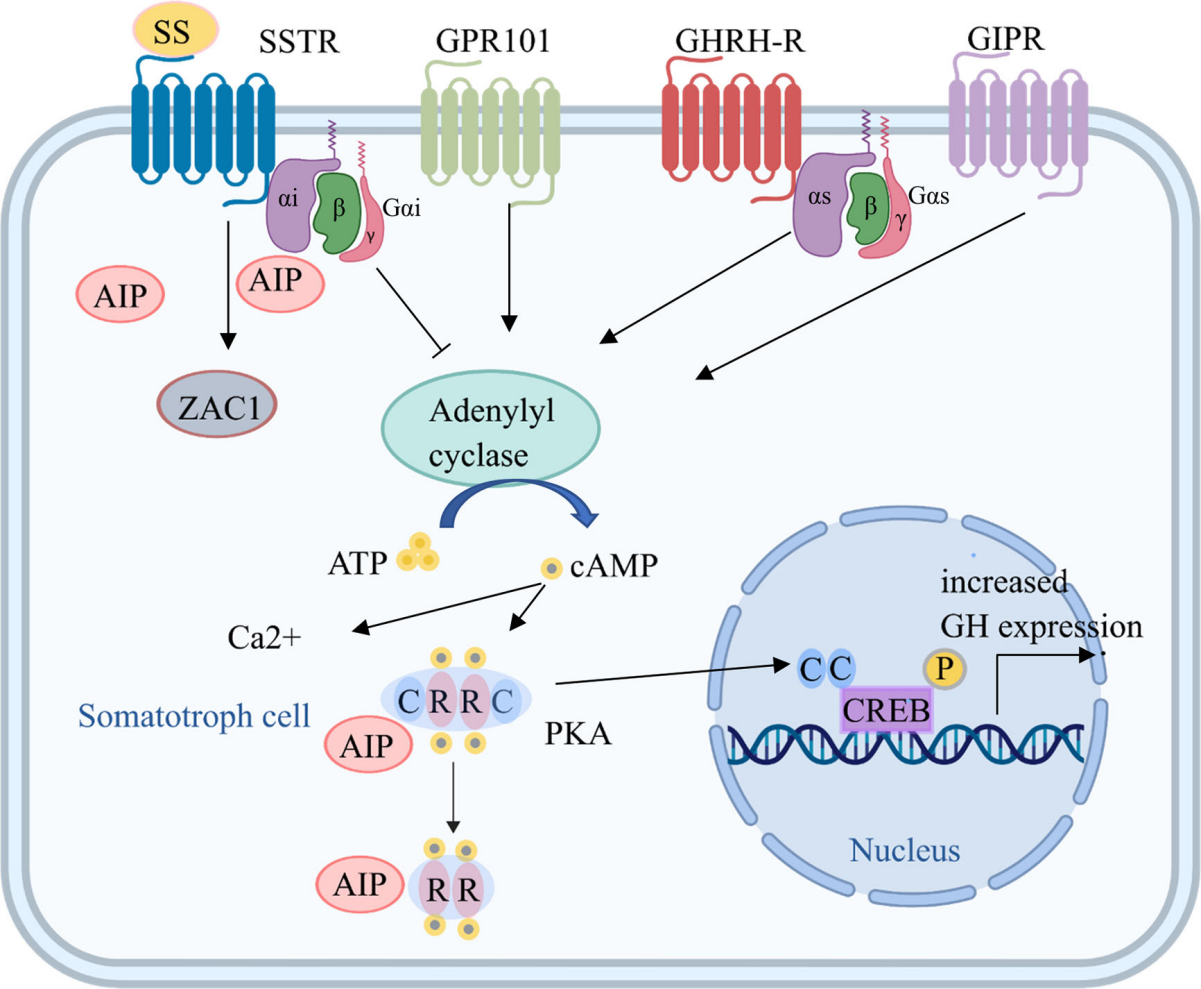
Tumorigenic mechanisms in somatotroph cells. Several mechanisms increase cAMP production, which is key for somatotroph tumorigenesis. Hormones bind to receptors, including GHRH-R, SSTR, GPR101, and GIPR, on the somatotroph cell membrane and increase the activation of adenylyl cyclase through Gsα. The consequent increase in cAMP production leads to the dissociation of the regulatory subunits of PKA from the catalytic subunits, which then translocate to phosphorylate CREB in the nucleus and other targets, leading to increased GH expression and cell proliferation. Gsα activation induced by *GNAS* mutations also leads to upregulation of the cAMP pathway. In addition, ectopic expression of GIPR may lead to an activated cAMP pathway, and GPR101 is a Gsα-coupled constitutively active receptor that leads to increased cAMP signaling. AIP, aryl hydrocarbon receptor-interacting protein; ATP, adenosine triphosphate; C, catalytic subunit; cAMP, cyclic adenosine monophosphate; CREB, cAMP response element; GHRH, growth hormone-releasing hormone; GHRH-R, GHRH receptor; GIPR, gastric inhibitory polypeptide receptor; GPR101, G protein-coupled receptor 101; Gsα, G protein stimulatory alpha subunit; GTP, guanine triphosphate; PKA, protein kinase A; R, regulatory subunit; SSTR, somatostatin receptor; ZAC1, zinc finger protein PLAGL1.

Somatotroph adenomas have greater genomic disruption than corticotroph adenomas or inactive tumors with no clinical evidence of hormone secretion (38). Gene mutations in PAs can be divided into heritable germline variations, mosaic mutations, and non-heritable somatic mutations (29). The former two are often familial and associated with syndromes, while the latter are sporadic (29). Mutations in aryl hydrocarbon receptor-interacting protein (*AIP*), *GNAS*, and cadherin-related 23 (*CDH23*), which are all involved in cAMP-associated pathways, are key for somatotroph tumorigenesis (**Figure 1**, **Table 1**).

**Table 1:** Genes affected in different pituitary tumors *via* genetic mutation or DNA methylation.

Gene (symbol)	Gene name	Location	Function of gene product and mechanism of tumorigenesis	Tumor types associated with each genetic defect
MEN1	Multiple Endocrine Neoplasia type 1	11q13.1	Tumor suppressor; Involved in cell proliferation, genome stability and gene transcription	Lactotroph adenomas, somatotroph adenoma, corticotroph adenomas, and nonsecreting adenomas
CDKN1B	cyclin-dependent kinase inhibitor 1B	12p13.1	Cell cycle regulation	Prone to developing somatotroph adenomas, and can also develop other types of PAs
PRKAR1A	Protein Kinase CAMP-Dependent Type I Regulatory Subunit Alpha	17q24.2	Loss of PRKAR1A causes enhanced PKA signaling.	Somatotroph adenomas and lactotroph adenomas
GPR101	G-protein-coupled receptor gene	Xq26.3	G-protein-coupled receptor; defects lead to constitutive activation of the cAMP-PKA pathway	Somatotroph adenomas
SDHx	Succinate dehydrogenase x	/	Unknown	More likely to produce prolactin; pituitary hyperplasia in mice
VHL	Von Hippel–Lindau	3p25.3	likely by modulating vascular endothelial growth factor expression and inducing apoptosis	Pituitary stalk hemangioblastomas
DICER	Dicer 1, Ribonuclease III	14q32	Unknown	Pituitary blastoma
MLH1	MutL Homolog 1	3p22.2	Unknown	Corticotroph adenomas
MSH2	MutS Homolog 2	2p21	Unknown	Corticotroph adenomas
AIP	Aryl hydrocarbon receptor interacting protein	11q13.2	Interaction in cAMP synthesis	All types
GNAS	Guanine nucleotide activating subunit	20q13.32	cAMP-regulating protein Gsα; activation leads to increased cAMP levels and activation of protein kinase A (PKA)	Mainly in somatotroph adenomas
PTTG1	Pituitary tumor-transforming gene-1	5q33.3	Enhanced PTTG1 is a regulator of sister chromatid segregation, this may subsequently drive chromosomal instability	All types
STAT3	Signal Transducer and Activator of Transcription 3	17q21.2	Enhanced STAT3 increased GH transcription.	Somatotroph adenomas
CDH23	Cadherin related 23	10q22.1	Calcium-dependent cell-cell adhesion glycoprotein	Somatotroph adenomas account for the highest proportion
IGSF1	Immunoglobulin superfamily member 1	Xq26.1	IGSF1 mutation weakens its transport to the cell surface in allogenic cells and increased total GH secretion and IGF-1 levels	Hyperplasia with increased total GH secretion and IGF-1 levels
SLC20A1	Solute Carrier Family 20 Member 1	2q14.1	Increased expression of SLC20A1 may be associated with the activation of the Wnt/β-catenin signaling pathway	Somatotroph adenomas
PRDM2	PR/SET Domain 2	1p36.21	The absence of PRDM2 involved the tumorigenesis through regulating c-Myc	Somatotroph adenomas
SSTRs and DRDs	Somatostatin receptors (SSTR1-5) and dopamine receptors (DRD1-5)	/	Decreased expression of receptors (DRD4, DRD5, SSTR1 and SSTR2) may be associated with poor response to SSAs	Somatotroph adenomas and silent somatotroph adenomas
IGSF1	Immunoglobulin Superfamily Member 1	Xq26.2	Membrane glycoprotein with modified residue possibly altering interaction with an extracellular ligand	Hyperplasia, and sometimes with GH secretion and IGF-1 level increasing
SLC20A1	Solute Carrier Family 20 Member 1	2q14.1	Increased SLC20A1 expression may be associated with activation of the Wnt–β-catenin signaling pathway	Somatotroph adenomas
PRDM2	PR domain zinc finger protein 2	1p36.21	c-Myc regulation	Somatotroph adenomas
GADD45γ	Growth Arrest and DNA Damage Inducible Gamma	9q22.2	Tumor suppressor; Involved in DNA damage and function in the negative regulation of cell growth	Non-secreting adenomas and somatotroph adenomas
LGALS3	Galectin 3	14q22.3	Promoter methylation status of LGALS3 for the regulation of Gal-3 expression in PA	Lactotroph adenomas, corticotroph adenomas
RASSF1A	Ras Association Domain Family Member 1	3p21.31	Promoter methylation of RASSF1A is detected in all types of PAs and mechanism is unknown,	All types
USP8	Ubiquitin Specific Peptidase 8	15q21.2	Involved in deubiquitination of EGFR; gain of functions mutations results in increased EGFR, and POMC expression	Corticotroph adenomas
USP48	Ubiquitin Specific Peptidase 48	1p36.12	Deubiquitination; activation of MAPK and increased POMC expression	Corticotroph adenomas
BRAF	B-Raf Proto-Oncogene, Serine/Threonine Kinase	7q34	Proto-oncogene with tyrosine kinase activity; activation of MAPK and increased POMC expression	Corticotroph adenomas
USP90	Heat Shock Protein 90	/	Unknown	Corticotroph adenomas
HDAC2	histone deacetylase 2	6q21	Unknown	Corticotroph adenomas
CABLES1	Cdk5 And Abl Enzyme Substrate 1	18q11	Unknown	Corticotroph adenomas
SFRP2	Secreted Frizzled-Related Protein 2	4q31.3	overexpression of SFRP2 in AtT20 cells reduces β-catenin levels in the cytoplasm and nucleus, and also decreases Wnt signaling activity	Corticotroph adenomas
POMC	Proopiomelanocortin	2p23.3	Unknown	Corticotroph adenomas
FGFR2	Fibroblast Growth Factor Receptor 2	10q26.13	Inducing Rb phosphorylation and regulation of cell cycle progression by p21 and p27	Corticotroph adenomas
PTAG	Pituitary Tumor Apoptosis Gene	22q12.2	Unknown	Corticotroph adenomas
TSP-1	Thrombospondin-1	15q14	Unknown	Corticotroph adenomas
CASP-8	Caspase-8	2q33.1	Unknown	Corticotroph adenomas
CABLES1	Cdk5 And Abl Enzyme Substrate 1	18q11	Unknown	Corticotroph adenomas
C5orf66-AS1	C5orf66 Antisense RNA 1	5q31.1	Unknown	Pituitary null cell adenomas
IL-6R, JAK2, STAT3, p-STAT3, and MMP9	Interleukin 6 receptor/Janus kinase 2/STAT3/matrix metallopeptidase 9	/	Unknown	Pituitary null cell adenomas
PI3K	Phosphatidylinositol 3-kinases	3q26.32	Oncogene; Involved in PI3K/AKT pathway which regulates several cellular functions, including cell survival, growth, proliferation, and metabolism	Non-secreting adenomas
CDKN2A	Cyclin dependent Kinase Inhibitor 2A	9p21	Tumor supperessor; Cell cycle regulation (G1 to S phase transition)	Non-secreting adenomas and somatotroph adenomas
ENC1	Ectodermal-neural cortex 1	5q13.3	Unknown	Null cell adenomas
MEG3	Maternally expressed 3	14q32.2	Tumor supperessor; suppress tumor genesis by both p53-dependent and p53-independent pathways.	Non-secreting adenomas
ING2	Inhibitor of growth family member 2	4q35.1	Unknown	Non-secreting adenomas
FAM90A1	family with sequence similarity 90 member a1	12p13.31	Unknown	Non-secreting adenomas

#### Aryl Hydrocarbon Receptor-Interacting Protein

Familial isolated pituitary adenoma (FIPA) is characterized by the familial occurrence of PAs in the absence of other clinical features (39). Germline mutations in the *AIP* gene are detected in approximately 20% of FIPA families and 50% of familial acromegaly families (40–43). *AIP* is located on human chromosome 11q13.2, and acts as a tumor suppressor in PAs (44). Mutations in *AIP* have been identified as causing a predisposition for PAs of variable penetrance in 20% of FIPA families (41). *AIP* mutations are usually associated with somatotropinomas, but prolactinomas, non-functioning PAs (NF-PAs), Cushing’s disease, and other infrequent clinical adenoma types can also occur (40–43). *AIP* mutations are common in male pediatric acrogigantism patients, and tend to cause large and invasive tumors; densely granulated subtypes rarely occur, and patients with *AIP* mutations are often resistant to somatostatin analogue (SSA) treatment (42, 43, 45, 46). Families with *AIP* mutations show incomplete penetrance, of approximately 15–30% (40, 42, 47). Genetic screening can identify carrier family members, and clinical screening has been reported to result in the earlier recognition of clinically relevant disease in approximately 20% of patients (22/187) (43).

Mechanically, some of the mutations lead to truncation of the AIP protein and loss of the C-terminal sequence, which affects protein interactions and leads to disrupted AIP function. A number of mechanisms may explain the resistance of *AIP*-mutated patients to SSAs. First, one mechanism may involve the reduced expression of the inhibitory G protein subtype, Gαi-2, which mediates the inhibitory effects of SSAs (44). Second, AIP has been reported to interact with both phosphodiesterase (PDE) and guanine nucleotide-binding proteins (G proteins); PDE4 expression is lower in *AIP*-mutated PAs, and interactions between PDE4 and AIP are disrupted by such mutations (44, 48, 49). Consistent with these findings, both *PDE* isoforms are reportedly overexpressed in GH cells from sporadic *AIP* mutation-negative GH-secreting adenomas (49). Third, AIP interacts with the protein kinase A (PKA) complex. *AIP* mutations affect the PKA pathway, thus affecting cell proliferation and development and the inflammatory response (42, 49). Fourth, another mechanism of SSA resistance may be related to the SSTR2–zinc-finger protein 1 PLAGL1 (ZAC1) pathway (50). *AIP* is upregulated by SSAs, and *AIP* can in turn upregulate ZAC1 mRNA expression (51, 52). Disordered cAMP regulation is important for the resistance of *AIP* mutations to SSA treatment (42, 49).

#### Guanine Nucleotide-Binding Protein G(s) Subunit Alpha

*GNAS* encodes the stimulatory α subunit of the G protein complex, which plays an important role in transmembrane signal transduction (53). Its mutation rate is the highest of somatic mutations in somatotroph adenomas (up to 40%). *GNAS*-mutated tumors are often smaller and less invasive, respond better to SSAs, and are usually densely granulated somatotroph adenomas (52, 54, 55). In addition, *GNAS*-mutated tumors have relatively high expression of dopamine receptor (DRD) 2, which suggests a good response to dopamine agonists (38). Somatic mutations in *GNAS* can result in sporadic somatotroph adenomas, while mosaic mutations for codon 201 likely result in McCune–Albright syndrome. This syndrome is characterized by polyostotic fibrous dysplasia, skin hyperpigmentation, and autonomous endocrine hyperfunction (56).

*GNAS* mutations can also lead to disruption of the cAMP signaling pathway. Especially, mutations in codon 201 or 227 result in the inhibition of G proteins and the activation of adenylyl cyclase, promote cAMP synthesis in cells, and drive tumorigenesis (57). Recently, a *GNAS* mutation (p.Arg201Cys) has been detected as a recurrent somatic event, and this mutation is shared with chromosome losses (58, 59).

#### Copy Number Variations at the Chromosomal Level

Recent studies have indicated that increased cAMP in tumorigenesis can probably induce DNA damage, leading to somatic CNVs and genome instability (60). Of these, arm-level CNVs are the most common abnormalities in somatotroph adenomas. Specifically, whole chromosome losses (chromosomes 1, 6, 13, 14, 15, 16, 18, and 22) and gains (chromosomes 3, 5, 7, 10, 19, 20, and X) have been observed (58, 59, 61). Interestingly, *GNAS* mutation-positive adenomas have relatively low CNV levels, whereas *GNAS* mutation-negative adenomas have a high degree of genomic disruption (58, 59, 61). These CNVs likely affect the Ca^2+^ and ATP pathways, which are involved in PA tumorigenesis (58, 59, 61). Thus, the CNVs in *GNAS* mutation-negative somatotroph adenomas provide an alternative tumorigenic pathway, which is linked to genomic instability (58, 59, 61).

#### Less Common Genetic Variations Potentially Associated With Pituitary Adenomas

Pituitary tumor-transforming gene 1 (*PTTG1*) is overexpressed in various pituitary tumors, and its expression is higher in more aggressive tumors (62–64). Somatotroph adenomas with recurrent aneuploidy have relatively high expression of *PTTG1*; as a regulator of sister chromatid segregation, this may subsequently drive chromosomal instability (64–66).

#### Signal Transducer and Activator of Transcription 3

STAT3 is a member of the STAT family, and participates in cellular responses to cytokines and growth factors (67). Its expression is enhanced in somatotroph adenomas, leading to GH hypersecretion, which in turn promotes *STAT3* expression (68). In primary human somatotroph adenoma-derived cell cultures, the specific inhibitor S3I-201 can inhibit *STAT3* expression, thus decreasing GH transcription and reducing GH secretion (68).

#### CDH23

*CDH23* is involved in Wnt pathway regulation. The *CDH23* c.4136G>T (p.Arg1379Leu) mutation leads to an amino acid substitution in the calcium-binding motif of the extracellular cadherin domain, which is predicted to disrupt cell–cell adhesion. The incidence rate of *CDH23* mutations is 33% in FIPA patients and 12% in sporadic PA patients. Of the *CDH23*-mutated PAs, somatotroph adenomas account for the highest proportion (25.9%) (69). PAs with functional *CDH23* variants are smaller and less aggressive compared with non-mutated PAs (69, 70). In addition, variants in this gene are associated with Usher syndrome (70). However, *CDH23* variations have been reported in only one study, and functional validation studies are needed.

#### Immunoglobulin Superfamily Member 1

IGSF1 is a membrane glycoprotein. *IGSF1* mutations can weaken its transport to the cell surface in allogenic cells, resulting in a novel X-related syndrome. This syndrome is characterized by central hypothyroidism, macro-orchidism, and prolactin deficiency (71–73). It can also be associated with acromegaloid facial features, increased head circumference, and increased total GH secretion and IGF-1 levels. However, considering that patients present with hyperplasia rather than adenomas, these symptoms may be secondary to the failure of regulatory and feedback mechanisms.

#### Solute Carrier Family 20 Member 1

*SLC20A1* levels are positively associated with tumor size, invasive behavior, and recurrence in somatotroph adenomas. In addition, increased *SLC20A1* expression may be associated with activation of the Wnt–β−catenin signaling pathway (74).

#### PR Domain Zinc Finger Protein 2

*PRDM2*, a tumor suppressor, plays an important role in cancer and obesity, including PAs. The absence of PRDM2 is likely to be involved in the tumorigenesis of somatotroph adenomas by regulating c-Myc (75). However, this discovery remains to be validated by more investigation groups.

#### Somatostatin Receptors and Dopamine Receptors

SSTR (SSTR1–5) and DRD (DRD1–5) subtypes play critical roles in the regulation of hormone secretion (76). Decreased expression of some of these receptors (DRD4, DRD5, SSTR1, and SSTR2) may be associated with a poor response to SSAs (77). In particular, SSTR2 expression might be a good predictor of a patient’s response to SSAs (78).

### DNA Methylation

DNA methylation is the most frequently studied epigenetic phenomenon, in which alterations of CpG dinucleotides block the transcriptional mechanism and silence gene expression (79). Approximately 80% of CpG dinucleotides are methylated in the human genome throughout the lifespan, and nearly 70% of CpG islands are methylated at any time, suggesting a widespread regulatory scope of DNA methylation (80). Here, we summarize the methylated genes in somatotroph adenomas (**Table 1**).

Growth arrest and DNA damage-inducible gene (*GADD45γ*) is a negative regulator of cell growth that is involved in DNA damage repair. In one study, most PAs (22/33, 67%) did not have *GADD45γ* expression, and 57.6% (19/33) of PAs were detected with *GADD45γ* methylation; there was significantly associated between *GADD45γ* methylated tumors and tumors in which GADD45γ transcript was not expressed (18 of 22; 82%; P = 0.002) (81). The silencing of *GADD45γ* is likely to confer a selective growth advantage during PA tumorigenesis (82).

The importance of the promoter methylation status of *LGALS3* (the gene encoding galectin-3) for the regulation of galectin-3 expression in PA was confirmed by Ruebel et al. Ikaros, a factor with transcriptional functions and chromatin-remodeling properties that determine the fate of hypothalamic neuroendocrine and pituitary cell populations during development, may also contribute to the expression of galectin-3 (83).

The human Ras-association domain family 1A (*RASSF1A*) gene has been reported as frequently (38%, 20/52) hypermethylated in its promoter region in all types of PAs. *RASSF1A* promoter methylation is relatively low in gonadotroph cell adenomas, higher in the most aggressive adenomas, and potentially correlated with Ki-67 expression. Reduced expression of RASSF1A has been identified in 18 of 20 (90%) adenomas with hypermethylation of *RASSF1A* (84).

### miRNA Regulation

miRNA binding to the 3’ and 5’ untranslated regions and coding sequences of target RNA is a form of post-transcriptional modification that results in differential gene expression. miRNAs play an important role in various pathways in tumors.

#### High-Mobility Group AT-Hook 1/2 Regulation

Palumbo et al (85). reported that miRNAs that target HMGA, including miR-15, miR-16, miR-26a, let-7a, miR-196a2, and other miRNAs, are downregulated in PAs. These HMGA-targeting miRNAs have also been demonstrated to inhibit the proliferation of a somatotroph adenoma cell line (GH3) and promote pituitary tumorigenesis. Furthermore, D’Angelo et al. reported that downregulated miRNAs, including miR-326, miR-570, and miR-432, target HMGA1 and HMGA2; while miR-34b and miR-548c-3p target HMGA2; and miR-603 and miR-326 target E2F transcription factor 1 (E2F1). In addition, some miRNAs are downregulated in somatotroph adenomas, including miR-34b, miR-326, miR-432, miR-548c-3p, miR-570, and miR-603 (86). The long non-coding RNA (lncRNA) ribosomal protein SA pseudogene 52 (*RPSAP52*) is overexpressed in PAs. It promotes cell proliferation in a competing endogenous RNA (ceRNA)-dependent manner by competitively binding to miR-15a, miR-15b, and miR-16, and by upregulating the expression of HMGA1 and HMGA2 (87, 88).

#### Phosphatase and Tensin Homolog –Protein Kinase B Pathway

Downregulated and upregulated miRNAs sometimes work together. For example, the downregulation of miR-26b expression together with the upregulation of miR-128 suppresses colony formation ability and invasiveness, and regulates the activity of the PTEN–AKT pathway in somatotroph adenomas (85).

#### PTTG1 Regulation

Multiple miRNAs are associated with the increased expression of *PTTG1*. First, miR-338-3p is upregulated in invasive somatotroph adenomas, and probably mediates the increased expression of *PTTG1* (89). Second, miR-423-5p, which targets *PTTG1*, shows decreased expression in somatotroph adenomas, and inhibits the expression of *PTTG1* at both the mRNA and protein levels (90). Third, overexpression of miR-524-5p downregulates the expression of *PTTG1*-binding factor, which interacts with *PTTG1* to mediate downstream effects, and significantly attenuates proliferation, migration, and invasion *in vitro* (91).

## Corticotropin-Secreting Adenomas

**Figure 2:**
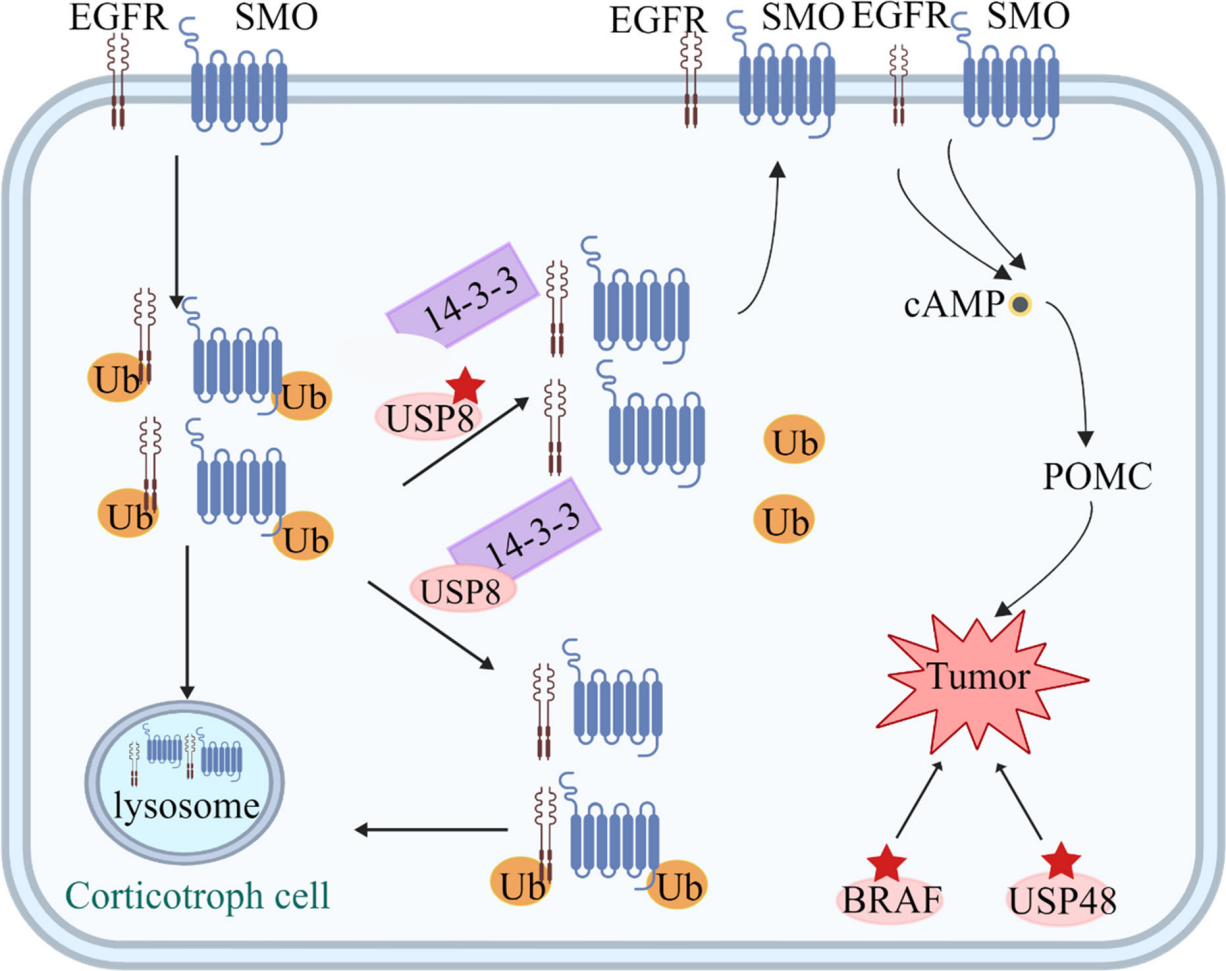
Tumorigenic mechanisms in corticotroph cells. USP8 removes ubiquitin tags from targets, such as EGFR and Smoothened (SMO), preventing them from undergoing proteasomal degradation and allowing recycling back to the cell surface. Increased EGFR and SMO activity leads to increased cAMP and POMC levels. Mutated USP8 cannot bind 14-3-3 protein and undergoes cleavage, which increase enzymatic activity, leading to increased deubiquitination of EGFR and SMO and higher expression of the two proteins on the cell membrane. *USP48* mutations and gain-of-function mutations of *BRAF* probably play a similar role to USP8.

Corticotropin-secreting adenomas account for 15% of PAs, with an incidence of 1.6 cases per 1 million individuals. These adenomas are typically small, and the excessive secretion of corticotropin leads to adrenal hypercortisolemia (92). Although 75% of patients achieve remission after surgical treatment, recurrence occurs in approximately 10% of these patients (93). Current pituitary-targeted drugs, including cabergoline and pasireotide, can improve the clinical features of excessive hormone secretion, but 60–75% of patients are insensitive to these drugs, which cannot control the symptoms long after drug treatment (94). Therefore, an understanding of the molecular characteristics of these tumors is necessary to identify additional drug targets. Here, we summarize the abnormal alterations in corticotropin-secreting adenomas that may serve as potential therapeutic targets (**Figure 2**, **Table 1**).

### Genetic Variations

#### Ubiquitin-Specific Peptidase 8

*USP8* encodes a deubiquitinase enzyme that protects epidermal growth factor receptor (EGFR) from degradation. Up to 62.4% of corticotropin-secreting adenomas were found to have *USP8* mutations that block 14-3-3 protein binding, leading to increased activity of USP8 (95–97). Gain-of-function mutations in *USP8* increase the deubiquitination of EGFR, which inhibits its degradation, leading to the activation of EGFR signaling. This mechanism likely leads to the synthesis and secretion of adrenocorticotropic hormone (ACTH) and promotes tumorigenesis (95–98). In *USP8*-mutated corticotropin-secreting adenoma samples, the cell cycle inhibitor p27, heat shock protein 90 (HSP90), and phosphorylated cAMP-response element binding protein (pCREB) were significantly reduced, suggesting that these proteins are direct or indirect clients of *USP8* and could therefore be potential targets for treatment (99).

*USP8* mutations have also been identified in silent corticotroph adenomas (100). Transcriptomic profiles show significant differences between functioning and silent corticotroph adenomas. However, *USP8* mutations have pleiotropic effects in both functioning and silent corticotroph adenomas, and affect the expression levels of many genes that are involved in a range of different pathways (100).

Germline *USP8* mutations, which are commonly found as somatic mutations in corticotropin-secreting adenomas, have also recently been described in a child with dysmorphic features, developmental delay, and a corticotroph adenoma (101). In addition, an overall prevalence of *USP8* mutations of 32% has been reported in corticotropin-secreting adenomas. *USP8*-mutated tumors are more common in females, and are associated with earlier onset (96, 102), a smaller size (95), and increased ACTH production (95, 102). Patients with *USP8*-mutated tumors are more likely to go into initial remission after surgery, but may also be more likely to show recurrence later in the clinical course (102–104). In contrast, *USP8*-mutation-negative tumors are more likely to show sphenoid invasion with an increased epithelial–mesenchymal transition signature (38). Lapatinib, an EGFR inhibitor, decreases proliferation *in vitro* and reduces tumor weight *in vivo* (105). In addition, SSTR5 expression is higher in *USP8*-mutated tumors (38, 106), potentially allowing mutation status to be used as a predictor of response to pasireotide (a second-generation SSA with a greater affinity for SSTR5) (107).

#### USP48 and BRAF

With the recognition of the importance of *USP8* mutations, another two mutated genes in the mitogen-activated protein kinase (MAPK) pathway, *USP48* and *BRAF*, have also been detected in corticotropin-secreting adenomas (108). Missense mutations in *USP48* include M415I/V substitutions, while V600E is an activation mutation of *BRAF*. Among tumors without *USP8* mutations, 23% of corticotropin-secreting adenomas have *USP48* mutations and 16% have *BRAF* mutations (108). Both mutations enhance the promoter activity and transcription of the ACTH precursor, the proopiomelanocortin (*POMC*) gene, and are potential therapeutic targets for the excess secretion of ACTH in corticotropin-secreting adenomas. Furthermore, *USP48* variants are associated with smaller tumors and a better response to corticotropin-releasing hormone (CRH) stimulation (109). Therefore, variations in *EGFR* regulation, the MAPK pathway, and *POMC*-related genes play a certain role in corticotropin-secreting adenomas.

#### Less Common Genetic Variations Potentially Associated With Pituitary Adenomas

##### Heat Shock Protein 90 (HSP90**)

Corticotroph adenomas overexpress HSP90 compared with the normal pituitary gland. N- and C-terminal HSP90 inhibitors act at different stages of the HSP90 catalytic cycle to regulate corticotropic cell proliferation and glucocorticoid (Gc) receptor (GR) transcriptional activity. The C-terminal HSP90 inhibitor silibinin has been reported to have anti-tumorigenic effects, partially decrease hormonal secretions, and alleviate the symptoms of Cushing’s disease in a mouse model (110).

##### Brg1 and Histone Deacetylase 2

The negative feedback regulation of *POMC* by GRs is a critical feature of the hypothalamo–pituitary–adrenal axis. Loss of Brg1 or HDAC2 should therefore produce Gc resistance, and we have previously shown that approximately 50% of Gc-resistant human and dog corticotropin-secreting adenomas, which are the hallmark of Cushing’s disease, have deficient nuclear expression of either of these proteins. In addition to providing a molecular basis for Gc resistance, Brg1 and HDAC2 deficiencies may also contribute to the tumorigenic process (111).

##### CDK5 and ABL1 Enzyme Substrate 1

CABLES1 is a cell cycle regulator that participates in the adrenal–pituitary negative feedback loop. Four heterozygous germline missense variants have been identified in *CABLES1* in four sporadic patients from a cohort of 182 patients with corticotropin-secreting adenomas, with functional evidence. The four variants affected residues within or close to the predicted cyclin-dependent kinase-3 (CDK3)-binding region of the CABLES1 protein and impaired its ability to block cell growth in a mouse corticotropinoma cell line (AtT20/D16v-F2). However, further studies are needed to assess the prevalence of *CABLES1* mutations in patients with other types of PAs, and to elucidate the pituitary-specific functions of this gene (111).

##### Secreted Frizzled−Related Protein 2 (SFRP2)

The RNA and protein expression of *SFRP2* is decreased in corticotroph adenomas compared with normal pituitary glands (112). In addition, the overexpression of *SFRP2* in AtT20 cells reduces β−catenin levels in the cytoplasm and nucleus, and also decreases Wnt signaling activity. *SFRP2* may therefore act as a tumor suppressor in Cushing’s disease by regulating Wnt signaling pathway activity. Clinically, there is an association between lower *SFRP2* expression and aggressive adenoma characteristics, including a larger size and invasiveness (112).

### DNA Methylation

Although some corticotropin-secreting adenomas have been found to have genetic mutations, the pathogenesis of non-mutated adenomas remains unknown. DNA methylation, a complementary mechanism for gene mutations, also plays an important role in PAs.

#### POMC

Hypomethylation of the promoter of *POMC*, which encodes the precursor of ACTH, leads to the occurrence of corticotropin-secreting adenomas. A comparison of the methylation profiles of ACTH-PAs and NF-PAs showed that the overexpression of *POMC* likely accounts for promoter hypomethylation (3). As a pituitary hormone, ACTH leads to increased serum cortisol levels in patients with Cushing’s disease, which is associated with the occurrence of corticotropin-secreting adenomas.

#### Fibroblast Growth Factor 2

FGF2 is a potent growth factor that regulates stem cell maintenance and neurogenesis during embryonic development and in response to challenges such as stress or injury in the adult brain (113–117). *FGFR2* encodes a growth factor receptor, and was found to be methylated by a 5’ promoter in mouse AtT20 cells, leading to significantly downregulated expression (80).

#### Pituitary Tumor Apoptosis Gene

In a model pituitary tumor cell line (AtT20), enforced expression of PTAG is associated with significantly increased sensitivity to the apoptotic effects induced by bromocriptine challenge (118).

#### Thrombospondin-1

The secreted angioinhibitory factor TSP-1 is an adhesive glycoprotein that mediates cell-to-cell and cell-to-matrix interactions, and is associated with platelet aggregation, angiogenesis, and tumorigenesis. Overexpression of TSP-1 in a murine AtT20 pituitary corticotroph tumor cell line leads to increased ACTH secretion (119).

#### ESR1 and Caspase 8

The methylation levels of *ESR1* and *CASP8* in corticotropin-secreting adenomas are also increased compared with those of silent corticotroph adenomas. *CASP8* plays an important role in apoptosis. Furthermore, *CASP8* has been suggested to affect the functional behavior of corticotroph adenomas, but the specific mechanisms remain unknown. The methylation of *POMC*, *FGFR2*, *RASSF1A*, *LGALS3*, and apoptosis-related factors plays a potential role in the development of corticotropin-secreting adenomas.

### MiRNA Regulation

The tumor suppressors miR-15a, miR-16, and miR-132 are downregulated in corticotroph adenomas. These miRNAs inhibit the proliferation, invasion, and migration of pituitary tumor cells by targeting sex-determining region Y-box 5 (SOX5) (120). In addition, the following miRNAs are also reportedly downregulated in corticotroph adenomas: miR-145 (2.0 fold), miR-21 (2.4 fold), miR-141 (2.6 fold), let-7a (3.3 fold), miR-150 (3.8 fold), and miR-143 (6.4 fold), suggesting that they may play a role in the tumorigenesis of corticotropin-secreting adenomas. miR-26a is overexpressed in corticotroph adenomas, and one of its direct targets is protein kinase Cδ (*PRKCD*) (85). *PRKCD* silencing is associated with increased EGFR expression, indicating PRKCD as a possible molecular target for the treatment of corticotroph adenomas (121, 122).

## Non-Secreting Adenomas

Non-secreting adenomas account for 20–35% of PAs originating from different hormone-secreting cells. Many of these are gonadotroph adenomas, and some show somatotroph, corticotroph, thyrotroph, or lactotroph differentiation. In older studies, the diagnosis of non-functional adenomas was mainly based on the normal results of hormone secretion rather than the results of immunohistochemical staining. However, with recently updated pituitary tumor classifications, the diagnosis of non-functional adenomas is made according to detailed pituitary cell types. Because excessive hormone levels are not found in the blood with non-secreting adenomas, they may not be detected for years in patients, and are often diagnosed incidentally (92). Although mostly benign, some invasive pituitary tumors require adjuvant radiation after surgery, often resulting in pituitary failure and a risk of recurrence. In an effort to aid in the development of additional treatment methods, we herein summarize the molecular signatures of non-secreting adenomas (**Table 1**).

### Genetic Variations in Null Cell Adenomas

#### C5orf66-AS1

*C5orf66-AS1* encodes a lncRNA that is differentially expressed between pituitary null cell adenoma tissues and normal pituitary tissues, as well as between invasive and non-invasive tumors (123). Co-expression analysis in RNA sequencing data revealed that *PAQR7* [a membrane progesterone receptor that may mediate a reduction in gonadotropin-releasing hormone in the progesterone negative feedback action in a progesterone receptor (A/B)-independent way (124)] was the gene that was most correlated with *C5orf66-AS1*, and several predicted trans-acting target genes, including *SCGB3A1* (encoding secretoglobin family 3A member 1), were also highly correlated with *C5orf66-AS1 *(123). These results indicate that *C5orf66-AS1* suppresses the development and invasion of pituitary null cell adenomas (123).

#### Interleukin 6 Receptor (IL-6R)/Janus Kinase 2 (JAK2)/STAT3/Matrix Metallopeptidase 9

Integrative proteomics and transcriptomics have revealed the activation of IL6R, JAK2, and STAT3, and the overexpression of IL-6R, JAK2, STAT3, p-STAT3, and MMP9, in invasive pituitary null cell adenomas (125). Therefore, activation of the IL-6R–JAK2–STAT3–MMP9 signaling pathway is correlated with the invasiveness of pituitary null cell adenomas.

### Genetic Variations in Silent Somatotroph Adenomas

#### Somatostatin Receptor 2 and Dopamine Receptor2

There is a negative correlation between *SSTR2* and tumor size in silent somatotroph adenomas. Additionally, levels of *DRD2* expression are reportedly similar between silent and functioning somatotroph adenomas, suggesting a possible basis for the treatment of these tumors with SSAs and dopamine (126).

### Genetic Variations in Non-Secreting Tumors Without Classification

#### Phosphatidylinositol 3-Kinases

PI3K is an important regulator of cell growth, transformation, adhesion, apoptosis, survival, and movement. The catalytic subunit encoding the *PIK3CA* gene is located on chromosome 3q26.3 and is often found to be deficient in cancer (127). Somatic mutations have been detected in 8 out of 91 (9%) of invasive pituitary tumors versus 0 out of 262 (0%) non-invasive tumors (128). In addition to mutations of *PI3K*, mutations of *MEN1*, *AIP* (129), and *CDH23* (69) have also been detected in non-secreting adenomas. In this section, we summarize the gene mutations in cell cycle regulators, phosphokinase, the cAMP signaling pathway, and cadherin that are associated with the occurrence of non-secreting adenomas.

### DNA Methylation

The p16 protein, encoded by the tumor suppressor gene *CDKN2A*, is located on chromosome 9p21. *CDKN2A* has been found to exhibit deletions, point mutations, or methylation inactivation in a variety of tumors. Methylation of the CpG island of *CDKN2A* was detected in 32/46 (70%) non-secreting adenomas, in contrast to 2/21 (9.5%) somatotroph adenomas and 0/15 histologically normal postmortem pituitaries (130). Methylation of *CDKN2A* corresponds to a loss of p16 expression on immunohistochemical analysis (130). When p16 is silenced, retinoblastoma protein (encoded by *RB1*) becomes phosphorylated, which enables cell cycle progression *via* the activation of E2F transcription factors (80). These observations indicate that the *CDKN2A*-related pathway is a potential target for non-secreting adenoma cell proliferation.

#### Maternally Expressed 3

*MEG3* is a maternally expressed imprinted lncRNA that is transcribed from multiple transcriptional variants with different splicing patterns, all of which are lncRNAs. Several studies have shown that *MEG3*-encoded lncRNA is a tumor suppressor that interacts with p53 and regulates expression of the p53 gene (*TP53*) (79, 131). In NF-PAs, *MEG3* was found to be hypermethylated in both the 5’ region of the first exon and approximately 1.6–2.1 kb upstream of the first exon, leading to the silencing of gene expression (79). Hypermethylation in the regulated area of *MEG3* is an important mechanism that likely leads to the loss of *MEG3* expression in clinical NF-PAs.

#### Ectodermal-Neural Cortex 1

Methylated *ENC1* has been found in tumor samples, and decreased levels were detected compared with normal pituitary glands (132). Notably, *ENC1* expression levels are reportedly lower in invasive null cell adenomas than in non-invasive adenomas (133, 134).

#### Inhibitor of Growth Family Member 2

*ING2* is a member of the growth inhibitor family, and participates in regulating the activity of histone acetyltransferase and histone deacetylase complexes, and plays a role in DNA repair and apoptosis. Methylation sequencing of NF-PAs revealed the presence of *ING2* DNA methylation in NF-PAs, and the methylation and expression levels were correlated with tumor recurrence. Furthermore, clinical data have indicated that *ING2* is an independent prognostic marker of NF-PAs (135).

#### Family with Sequence Similarity 90 Member A1

*FAM90A1* is a primate-specific gene that is associated with *ING2*. *FAM90A1* is also hypermethylated in NF-PAs, and the methylation level is significantly correlated with patient prognosis (135). *FAM90A1* methylation likely plays a role in NF-PA tumorigenesis and is a potential marker of PA.

#### Common Methylations with Somatotroph Adenomas

Similar to somatotroph adenomas, NF-PAs also exhibit hypermethylation in the CpG island of the *GADD45γ* gene. In addition, the methylation of *CDKN2A*, *MEG3*, and *RASSF1A* has also been detected in NF-PAs. Most methylated genes are involved in the cell cycle and DNA damage repair. However, novel abnormally methylated genes, such as *ING2* and *FAM90A1*, have also been detected, and their specific regulatory mechanisms remain unknown.

## Lactotroph Adenomas

Lactotroph adenomas are the most common secretory tumors, accounting for 60% of all PAs. Microprolactinomas are usually stable and slow growing, and continued growth after diagnosis occurs in less than 15% of cases (2). Lactotroph adenomas are ideally managed with dopamine agonists (136). Dopamine agonists can reduce prolactin levels and shrink tumors; however, they have side effects, and 15% of patients are not sensitive to these drugs (2). Additional targets are therefore needed to improve lactotroph adenoma treatment. Here, we summarize the molecular variations that occur in lactotroph adenomas.

### HMGA1 and HMGA2

HMGA proteins comprise a family of transcriptional regulating factors that are highly expressed during embryogenesis and play an important role in tumorigenesis in various tissues, including the pituitary gland. *HMGA2* has been shown to be rearranged and amplified in lactotroph adenomas, and transgenic mice with *Hmga2* overexpression develop PAs with prolactin and GH secretion (137). The primary mechanism by which *HMGA2* mutations lead to PAs involves an increase in E2F1 activity (138). It has also been reported that *MIA* is one of the most commonly downregulated genes in lactotroph adenomas, and this may serve as a downstream mechanism of *HMGA2* (137, 139). The HMGA2 protein can bind to the DNA elements of pituitary-specific positive transcription factor 1 (*PIT-1*) and cause a PIT-1 reaction, thus positively regulating the production of the *PIT-1* promoter. It is therefore speculated that HMGA2 leads to the proliferation of PIT-1-expressing cells or the abnormal proliferation of GH- and prolactin-secreting embryonic cells (137, 140). In addition, the downregulation of miRNAs may be related to the accumulation of HMGA2, which might lead to the proliferation of pituitary cells. In one study, the expression of miR-let-7 was reported to be decreased in 23 of 55 (42%) lactotroph adenomas, and was negatively correlated with the expression of HMGA2 (141). The overexpression of HMGA2 protein is also positively correlated with pituitary tumor phenotype, and overexpression of *Hmga2* in transgenic mice results in the development of PA (142). Therefore, *HMGA2* is a specific oncogene for pituitary transformation. However, according to the current evidence that has been collected on genes in the *HMGA* family, only *HMGA2* has a direct causal role in human pituitary tumorigenesis because it is amplified or rearranged in human lactotroph adenomas. It has been reported that *HMGA1* and *HMGA2* nuclear expression levels are significantly higher in invasive adenomas than in non-invasive adenomas (143, 144).

### Prolactin Receptor

The deletion of the PRLR-encoding gene leads to aggressive pituitary tumors in mice, while homozygous deletion mutants of *PRLR* in patients with hyperprolactinemia and agalactia do not lead to pituitary tumors (145). In contrast, a gain-of-function mutation was found in 9 of 46 patients with lactotroph adenomas, representing a potential novel mechanism for lactotroph adenoma tumorigenesis. In addition, three other rare variants and two low-frequency variants found in this cohort may represent benign changes (146). However, further investigations are needed to confirm the underlying mechanisms.

## Thyrotroph Adenomas

Thyrotroph adenomas are the least common type of PAs, accounting for approximately 1% of all adenomas. Thyrotroph adenomas lead to elevated or inappropriately suppressed thyrotropin levels, with normal or elevated thyroid hormone levels. Sapkota et al. confirmed six DNA variants as candidate driver mutations; two of these mutations were identified in genes with an established role in malignant tumorigenesis (*SMOX* and *SYTL3*), while four had unknown roles (*ZSCAN23*, *ASTN2*, *R3HDM2*, and *CWH43*) (147). Similarly, a single nucleotide polymorphism array analysis revealed frequent chromosomal regions of copy number gains, including recurrent gains at the loci harboring four of these six genes. In addition, Ando et al. reported a somatic mutation in the ligand-binding domain of thyroid hormone receptor beta (TRbeta) that causes a His to Tyr substitution at codon 435 of TRbeta1, corresponding to codon 450 of TRbeta2 (148). Unlike other PAs, thyrotroph adenomas are rarely associated with genetic syndromes or common somatic mutations.

## All Types

Gain or loss of chromosome. A non‐random gain in pituitary tumors has been reported in chromosomes 5, 8, 12, and X (149, 150). Gains of chromosomes 8 and 12 were found in prolactinomas and non-secreting adenomas, whereas a combined loss of chromosomes 5 and 8 was observed in corticotroph and somatotroph adenomas. In addition, recurrent structural rearrangements affecting chromosomes 1, 3, and 12 have also been identified in prolactinomas, which appear to be the only PA subtype with a defined trend of tumor‐specific chromosomal changes (149). In another whole-exome sequencing investigation, 75% of the highly disrupted group were functional adenomas or atypical null cell adenomas, whereas 87% of the less-disrupted group were non-functional adenomas (151). The disrupted samples were characterized by expression changes associated with poor outcomes in other cancers. Furthermore, arm-level losses of chromosomes 1, 2, 11, and 18 were significantly recurrent (151). These data indicate that sporadic PAs have distinct copy-number profiles that are associated with hormonal and histological subtypes and influence gene expression.

Transcription factors. Cell lineages of the pituitary are dependent on the expression of the pituitary transcription factors *PIT1*, *TPIT*, and *SF1* that, in concert with *ERα* and *GATA2/3*, regulate cellular differentiation and hormone secretion (152). Recently, these transcription factors (*PIT1* for GH, prolactin, and TSH lineages; *SF1* for gonadotroph lineages; and *TPIT* for ACTH lineages) have been added to the classification (152, 153).

DNA methylation. Frequently methylated genes (i.e., *CDKN2A*, *GADD45y*, *FGFR2*, *CASP8*, and *PTAG*) demonstrate methylation in over 50% of PAs; moderately methylated genes (i.e., *TSP-1*, *RASSF1A*, *RB1*, *p73*, *MGMT*, and *CDH1*) demonstrate methylation in 20–50% of PAs; and infrequently methylated genes (i.e., *p14*, *DAPK1*, *TIMP3*, *p21*, and *p27*) are methylated in less than 20% of PAs (79, 154). These variable rates of methylation may reflect a variety of factors.

Implications for therapy. In a subset of pituitary tumors that are refractory to routine therapy, both *MGMT* promoter hypermethylation in aggressive PAs and pituitary carcinomas and low protein expression have been implicated. Hypermethylation of *MGMT* indicates a better response to treatment with temozolomide (TMZ) in some aggressive PAs (155). In one study, combination treatment with TMZ and pyrimethamine (PYR) produced synergistic antitumor activity both *in vivo* and *in vitro.* Moreover, TMZ/PYR treatment induced cell cycle arrest, increased DNA damage, and upregulated the expression of cathepsin B, BAX, cleaved PARP, and phosphorylated histone H2AX; it also elevated caspase 3/7, 8, and 9 activities. Therefore, PYR may enhance the efficacy of TMZ by triggering both cathepsin B- and caspase-dependent apoptotic pathways. A combination of PYR and TMZ may thus provide a novel regimen for invasive PAs that are refractory to standard therapy and TMZ alone (156).

## Conclusion

We reviewed older and more recent discoveries of molecular variations in somatotroph adenomas, corticotroph adenomas, lactotroph adenomas, non-secreting adenomas, and thyrotroph adenomas. Summarizing the genetic and epigenetic markers of different types of PAs and elucidating their comprehensive molecular mechanisms will be helpful to identify the physiological pathways of PAs. These findings will provide a better understanding of the occurrence of PAs and lead to the development of novel therapeutic treatments.

## Author Contributions

CM wrote the manuscript of this paper, and YC helped in modifying the manuscript. BX and WR directed the manuscript. All authors contributed to the article and approved the submitted version.

## Funding

This work was supported by the National Key R&D Program of China (2018YFA0108602, 2018YFA0108600 to XB), China Postdoctoral Science Foundation (BX20190045 to MC), Beijing Municipal Natural Science Foundation (7182134 to XB), CAMS Initiative for Innovative Medicine (CAMSI2M) (2016-I2M-1-017 to XB), CAMS Young Talents Award Project (2018RC320003 to XB), and Beijing Nova Program (Z181100006218003 to XB).

## Conflict of Interest

The authors declare that the research was conducted in the absence of any commercial or financial relationships that could be construed as a potential conflict of interest.
